# Supplementary material for: Bone marrow endosteal stem cells dictate active osteogenesis and aggressive tumorigenesis
Source: Nat Commun. 2023 Apr 25;14:2383. doi: 10.1038/s41467-023-38034-2 (PMC10130060; doi:10.1038/s41467-023-38034-2)
Supplement: Supplementary file 1 — Supplementary Information [file 41467_2023_38034_MOESM1_ESM.pdf]

Supplementary Information

Bone marrow endosteal stem cells dictate active osteogenesis and aggressive tumorigenesis

Matsushita et al.

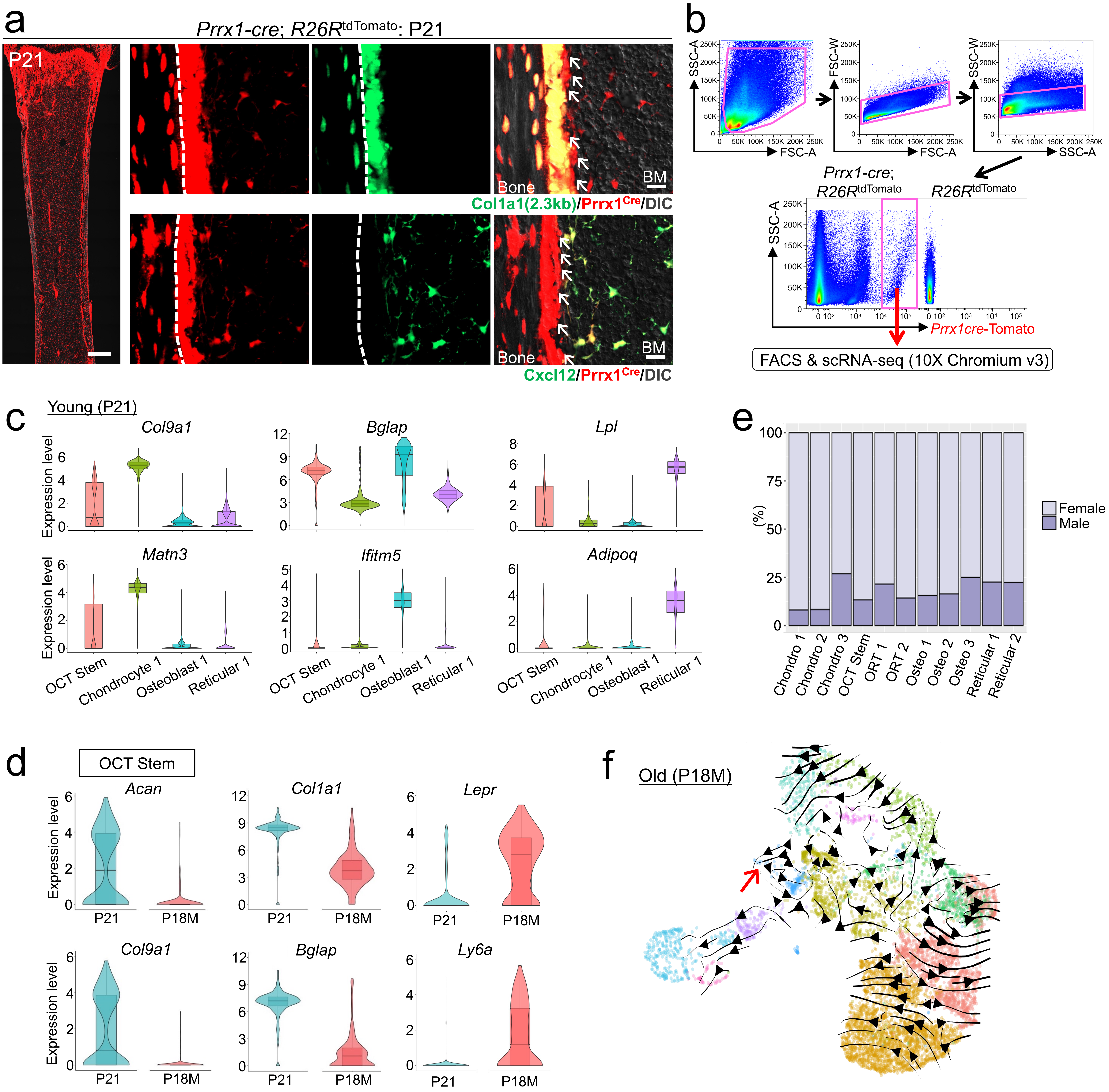

Supplementary Figure 1. Osteoblast-chondrocyte transitional (OCT) stem cells in young bone marrow.

(a) *Prrx1-cre*; *R26R*^{tdTomato} femur at P21 with growth plates on top (left, Scale bar: 500μm). Right: endosteal space in high magnification. *Prrx1-cre*; *R26R*^{tdTomato} femur with Col1a1-GFP (upper right 3 panels, scale bar: 20μm) and *Prrx1-cre*; *R26R*^{tdTomato} femur with Cxcl12-GFP (lower right 3 panels, scale bar: 20μm). Arrows: endosteal tdTomato^{+} cells without Col1a1-GFP or Cxcl12-GFP expression. Broken lines: bone surface. *n*=3 (*Col1a1-GFP*; *Prrx1-cre*; *R26R*^{tdTomato}), *n*=3 (*Cxcl12-GFP*; *Prrx1-cre*; *R26R*^{tdTomato}) mice.
(b) FACS-sorting of Prrx1^{cre}-tdTomato^{+} cells. Gating strategy for bone marrow cells isolated from *Prrx1-cre*; *R26R*^{tdTomato} mice at P21.
(c) Violin plots of representative chondrocyte *(Col9a1, Matn3)*, osteoblast *(Bglap, Ifitm5)* and reticular cell *(Lpl, Adipoq)* markers in representative clusters (OCT Stem, Chondrocyte 1, Osteoblast 1 and Reticular 1) at P21. *n*=570 (OCT Stem), *n*=764 (Chondrocyte 1), *n*=1,181 (Osteoblast 1), *n*=1,797 (Reticular 1) cells.
(d) Violin plots of representative positively enriched genes at P21 *(Acan, Col9a1, Col1a1, Bglap)* and at P18M *(Lepr, Ly6a)* in OCT Stem cluster.
(e) Percentage of female (grey) and male (purple) cells in each cluster of P21 Prrx1^{cre}-tdTomato^{+} dataset. *n*=570 (P21), *n*=219 (P18M) cells.
(f) RNA velocity analysis at P18M. Black arrows: dynamic velocity vectors inferring future states. Red arrows: OCT Stem cluster.
Data are presented as median (mid-line in the plot) [25 percentile (lower bound in the plot), 75 percentile (upper bound in the plot)] of the log2-transformed normalized gene expression.

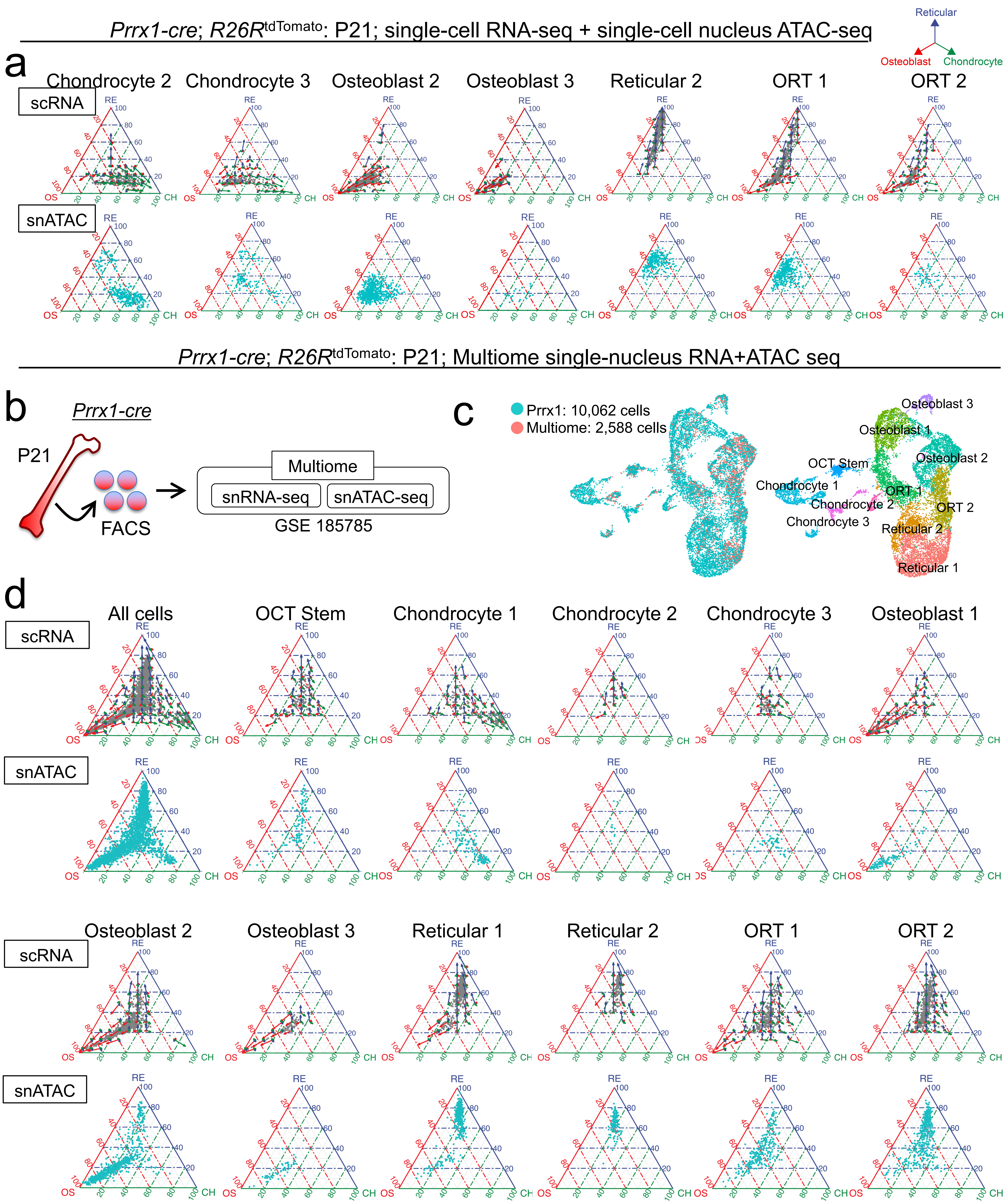

Supplementary Figure 2. Osteoblast-chondrocyte transitional (OCT) stem cells in young bone marrow.

(a) Simplex and velocity analyses of representative cell types from P21 Prrx1^{cre}-tdTomato^{+} scRNA-seq data. Cells colored in grey show their transcriptomic affinities towards three vertices whereas the arrows show the future differentiation potential of the cells falling in the corresponding bin. Cells colored in aqua show their epigenomic affinities towards three vertices. Blue arrow and axis: Reticular cluster. Red arrow and axis: Osteoblast cluster. Green arrow and axis: Chondrocyte cluster.

(b) Single-cell Multiome RNA+ATAC seq analyses of P21 Prrx1^{cre}-tdTomato^{+} cells.

(c) UMAP visualization of P21 Prrx1^{cre}-tdTomato^{+} scRNA-seq and P21 Multiome snRNA-seq data merged by LIGER. Cells were pooled from *n*=3 (scRNA-seq), *n*=3 (Multiome) *Prrx1-cre*; *R26R*^{tdTomato} mice. Left: UMAP plots colored by datasets with scRNA-seq as blue and Multiome as red. Right: UMAP plots colored by major sub-clusters of Chondro [Chondrocyte (1-3)], Osteo [Osteoblast (1-3)], Adipo [Reticular (1-2)], osteoblast-reticular transitional (ORT) cells (1-2) and osteoblast-chondrocyte transitional (OCT) Stem cells.

(d) Simplex and velocity analyses of single-cell Multiome RNA+ATAC seq data. Cells colored in grey show their transcriptomic affinities towards three vertices whereas the arrows show the future differentiation potential of the cells falling in the corresponding bin. Cells colored in aqua show their epigenomic affinities towards three vertices. Note that these single-cell multiome RNA + ATAC results are from experimentally measured cell correspondences. Blue arrow and axis: Reticular cluster. Red arrow and axis: Osteoblast cluster. Green arrow and axis: Chondrocyte cluster.

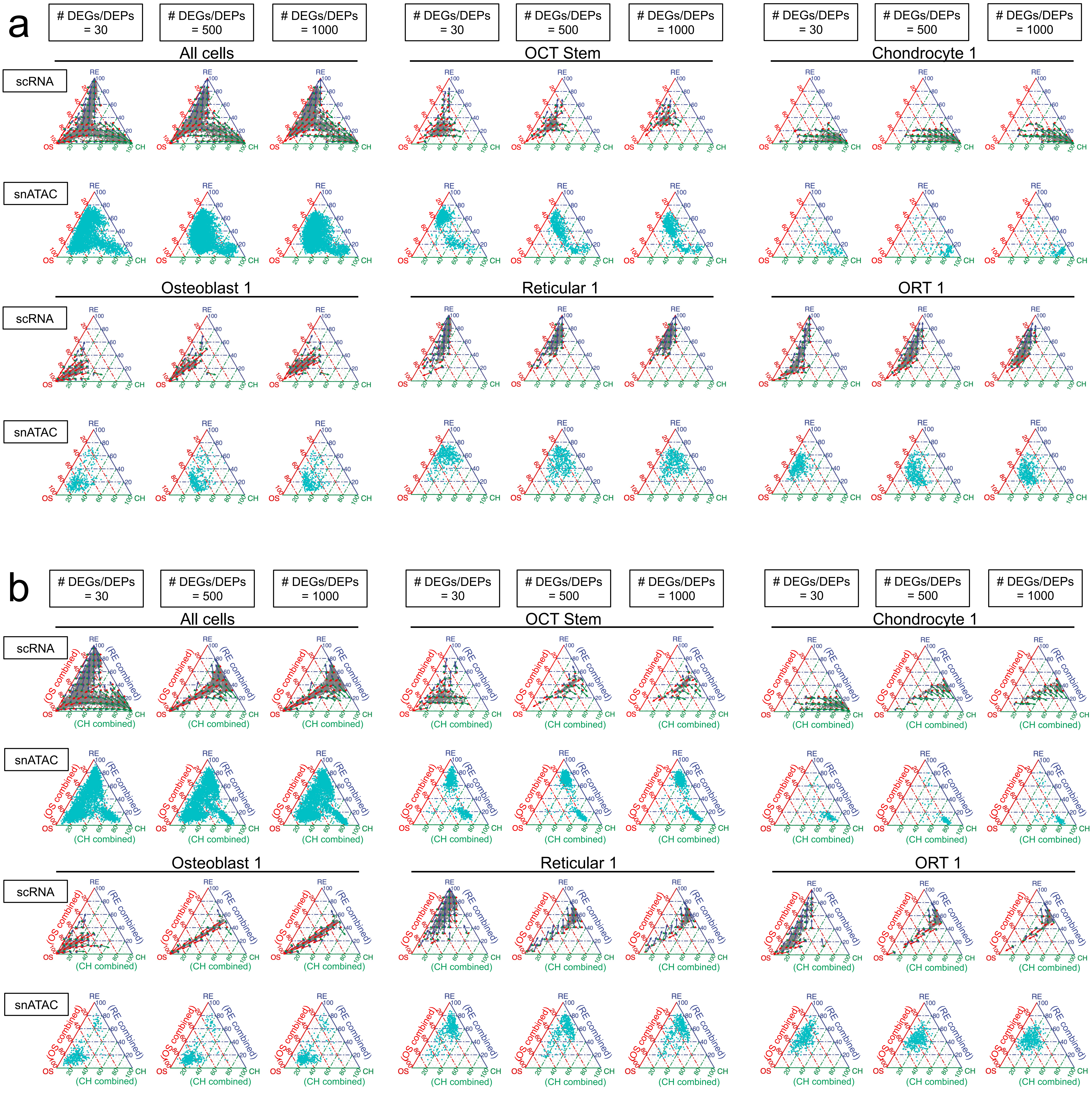

Supplementary Figure 3. Robustness of ternary plots using differentially expressed features and representative clusters in scRNA-seq and snATAC-seq data.

(a) Ternary plots of P21 scRNA-seq and snATAC-seq data are robust to the number of differentially expressed genes (DEG) or differentially accessible peaks (DAP) used, with a similar trend observed using 30, 500, and 1000 features.
(b) Using combined subclusters from each cell type as the reference introduces noise from other cell types, leading to distorted plots with every cluster becoming identical. The differentially expressed features were also derived from combined cell types.

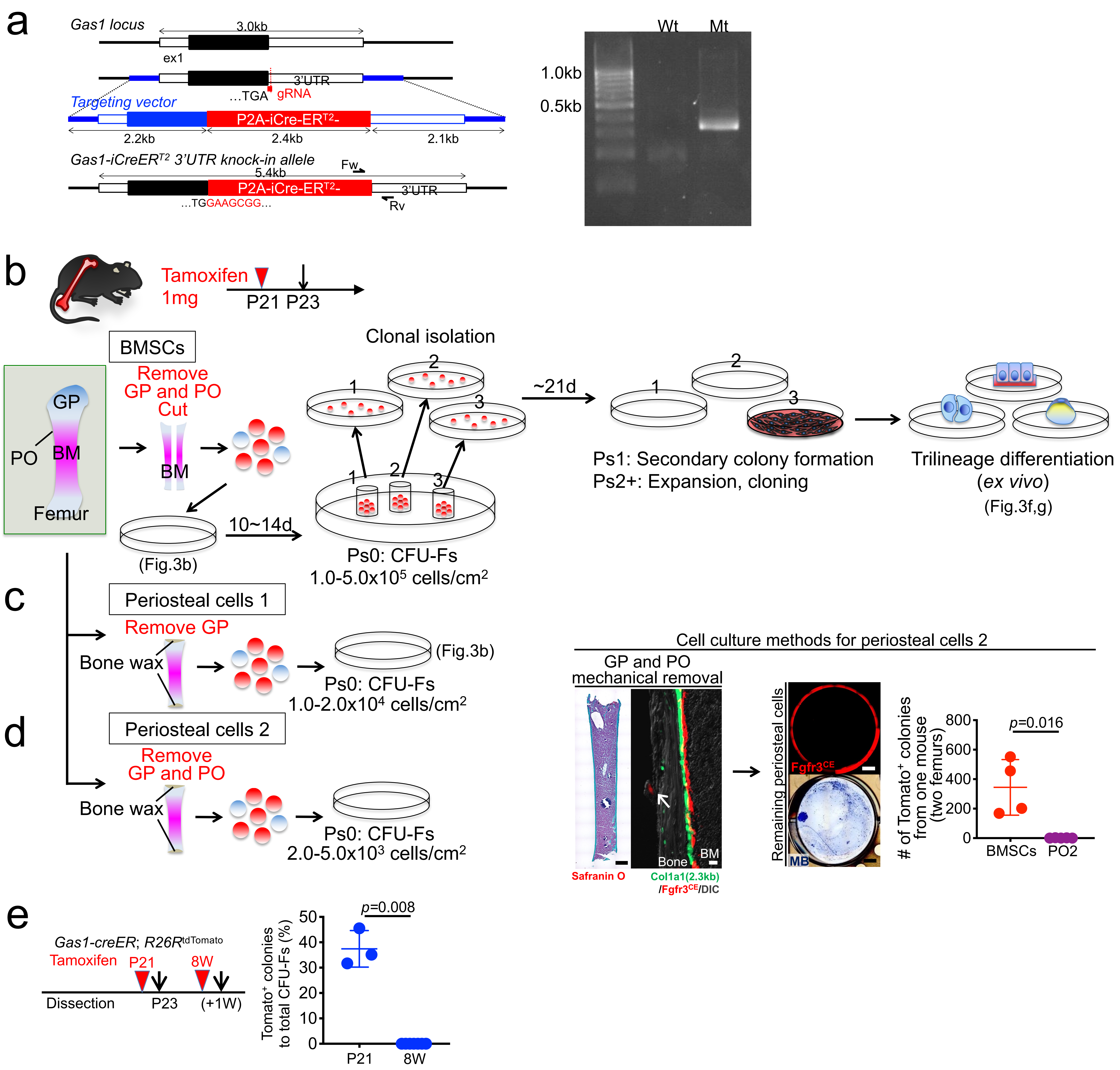

Supplementary Figure 4. Fgfr3^{+} endosteal stromal cells are enriched for skeletal stem cell activity.

(a) Structure of the genomic *Gas1* locus, targeting vector and knock-in allele after homologous recombination (left). White boxes: untranslated region; black boxes: coding regions; ex: exon. Blue bars: homologous arms; red bars: guide RNA as part of CRISP-Cas9 reagents; red boxes: P2A-iCreER^{T2}-bGHpA cassette replacing the native stop codon. Half arrows: primers, mutant forward and wild-type reverse. PCR genotyping using allele-specific primers (right). PCR genotyping was performed for all mice.
(b-d) Diagram of protocols to isolate *Fgfr3-creER*$^{+}$ cells from (b) bone marrow, (c) periosteum or (d) periosteal surface after mechanically removing periosteum. In (b), subsequent *in vitro* assays were designed to evaluate self-renewal and tri-lineage differentiation of individual Fgfr3-creER^{+} CFU-Fs. Primary colonies (Ps0: Passage 0) were isolated individually and further cultured independently (Ps1: Passage 1). Secondary colonies were passaged further (Ps2: Passage 2 and higher) and cultured under trilineage differentiation conditions *in vitro*. (d, right panels): CFU-F assays of periosteal cells isolated from periosteum-free femurs. Scale bar: 500µm (Safranin O), 20µm (fluorescent image). *n*=4 mice. (d, rightmost): Number of tdTomato^{+} colonies from one mouse / two femurs. Scale bar: 5mm. *n*=4 (Fgfr3^{CE}-bone marrow), *n*=5 (Fgfr3^{CE}-periosteum after removing periosteum mechanically) mice. Two-tailed, Mann-Whitney's *U*-test.
(e) Percentage of tdTomato^{+} colonies among total CFU-Fs across young and adult stages. Gas1^{CE} (pulsed at P21 or 8W) *n*=3 (P21), *n*=7 (8W). Two-tailed, Mann-Whitney's *U*-test.
Data are presented as mean ± s.d. Exact *P* value is indicated in the figures. Source data are provided as a Source Data file.

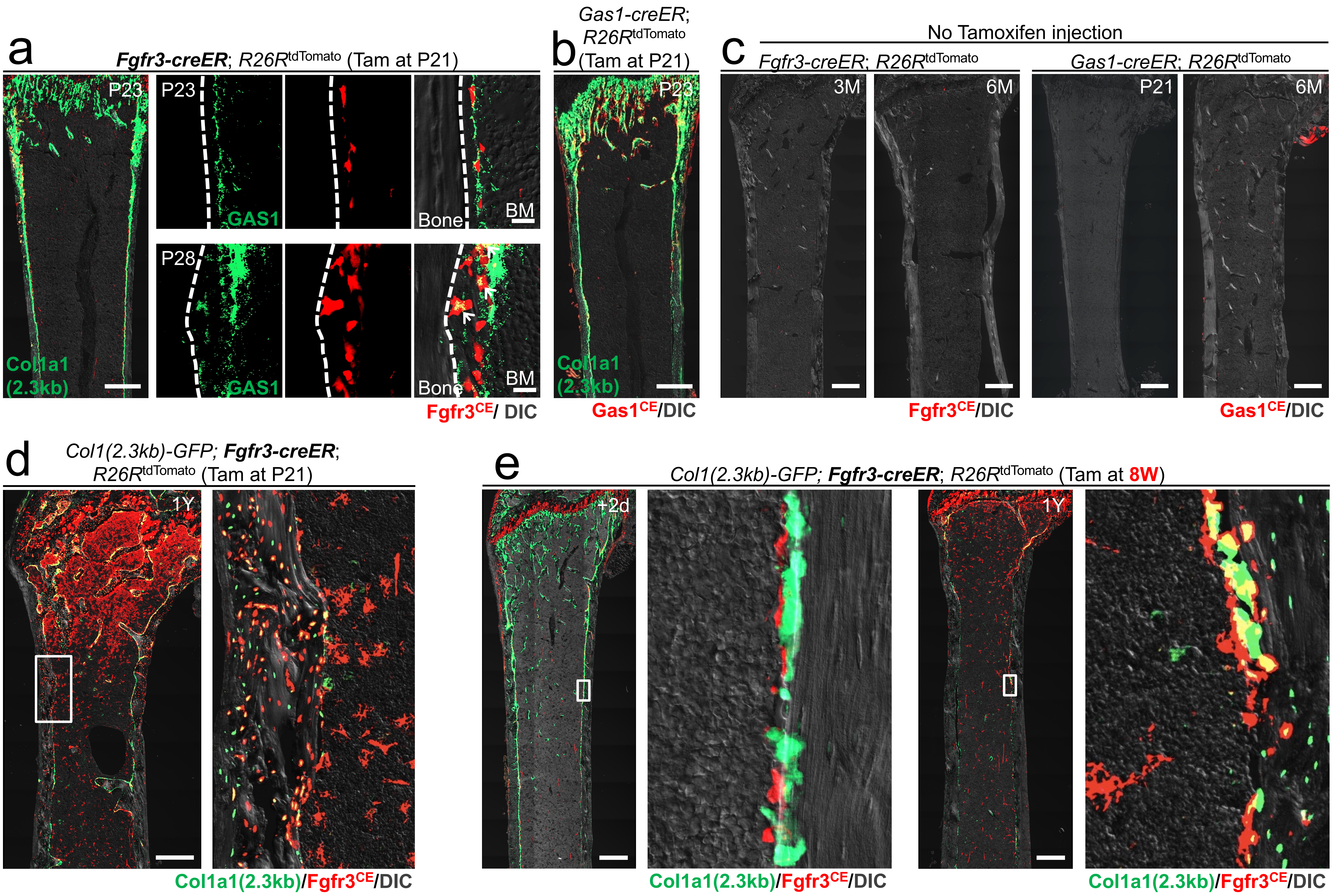

Supplementary Figure 5. Robust osteogenic capabilities of $Fgfr3^{+}$ endosteal stromal cells in homeostasis.

(a) *Fgfr3-creER*; $R26R^{tdTomato}$ femur at P23 and P28 (pulsed at P21) with Col1a1-GFP and GAS1 immunohistostaining. Scale bar: 500µm (leftmost), 20µm (others). *n*=4 mice.
(b) *Col1a1*-GFP; *Gas1-creER*; $R26R^{tdTomato}$ femur at P23 (pulsed at P21). Scale bar: 500µm. *n*=4 mice.
(c) No tamoxifen controls of *Fgfr3-creER*; $R26R^{tdTomato}$ femurs at 3M and 6M (left 2 panels), and *Gas1-creER*; $R26R^{tdTomato}$ femurs at P21 and 6M (right 2 panels). Scale bar: 500µm. *n*=3 mice per each group.
(d) *Col1a1-GFP*; *Fgfr3-creER*; $R26R^{tdTomato}$ distal femurs at 1Y (pulsed at P21). Scale bar: 500µm. *n*=4 mice.
(e) *Col1a1-GFP*; *Fgfr3-creER*; $R26R^{tdTomato}$ distal femurs after 2 days of chase (left two panels) and at 1Y (right two panels) (pulsed at 8W). Scale bar: 500µm. *n*=4 mice per each group.

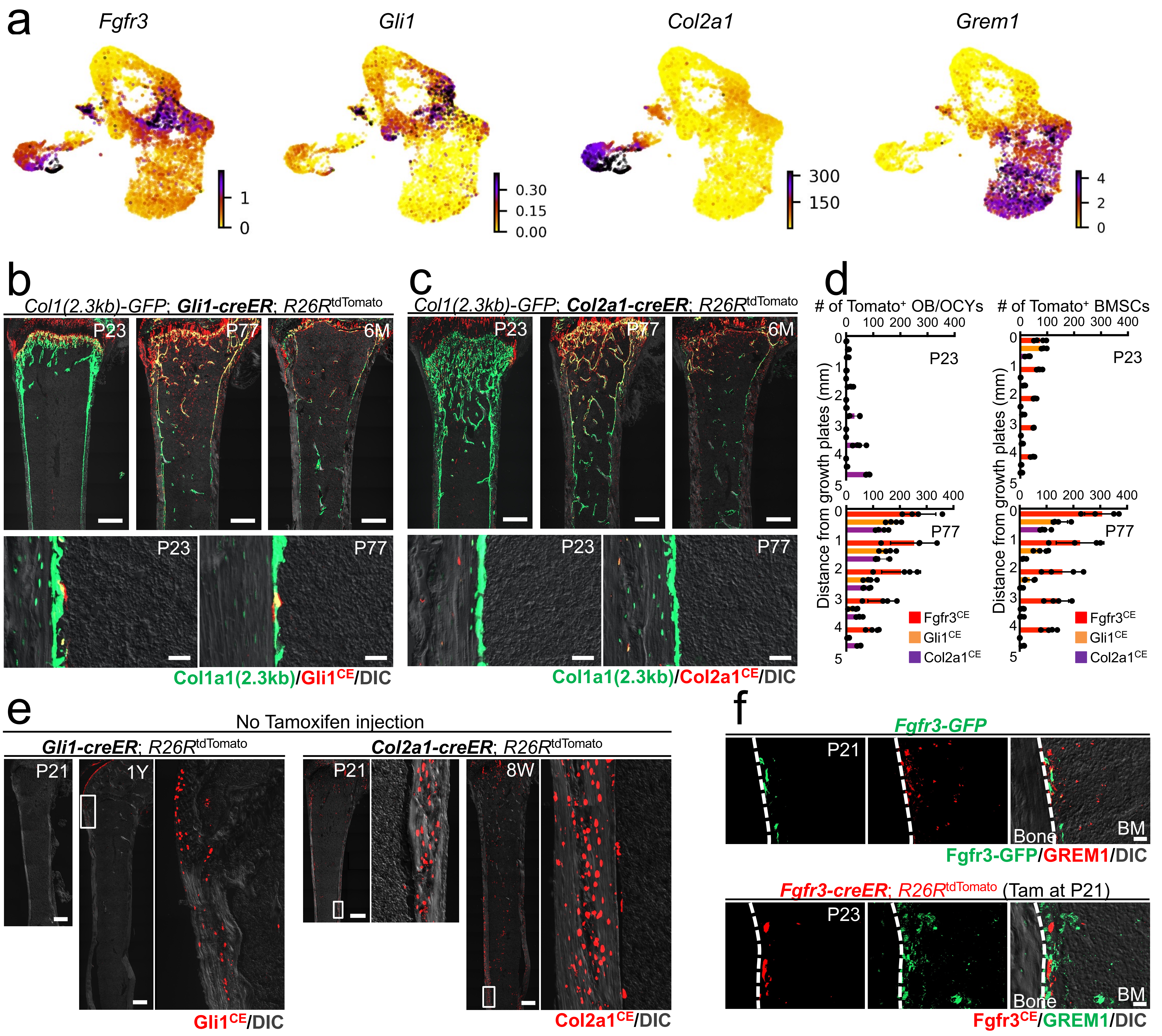

Supplementary Figure 6. Robust osteogenic capabilities of Fgfr3^{+} endosteal stromal cells in homeostasis.

(a) UMAP plots colored by expression of *Fgfr3, Gli1*, *Col2a1* and *Grem1*, P21 Prrx1^{cre}-tdTomato^{+} single-cell RNA-seq datasets. Violet: high expression, yellow: low expression.
(b) Long-chase analysis of Gli1-creER^{+} cells. *Col1a1-GFP; Gli1-creER; R26R^{tdTomato}* femur at P23 (upper left, lower left), P77 (upper center, lower right) and 6M (upper right) (pulsed at P21). Scale bar: 500µm (upper), 20µm (lower). *n*=4 mice per each time point.
(c) Long-chase analysis of Col2a1-creER^{+} cells. *Col1a1-GFP; Col2a1-creER; R26R^{tdTomato}* femur at P23 (upper left, lower left), P77 (upper center, lower right) and 6M (upper right) (pulsed at P21). Scale bar: 500µm (upper), 20µm (lower). *n*=4 mice per each time point.
(d) Quantification of tdTomato^{+} osteoblasts or osteocytes (left) and bone marrow stromal cells (right) in marrow space at P23 and P77, based on distance from growth plate. Red: Fgfr3^{CE}-tdTomato^{+}, Orange: Gli1^{CE}-tdTomato^{+}, Violet: Col2a1^{CE}-tdTomato^{+} cells. *n*=3 mice for Gli1^{CE} at P23, *n*=4 mice for Fgfr3^{CE} at P23 and P77, Gli1^{CE} at P77, and Col2a1^{CE} at P23 and P77.
(e) Tamoxifen-independent activities of Gli1-creER and Col2a1-creER in osteoblasts and osteocytes. Scale bar: 500µm. *n*=3 mice per each group.
(f) GREM1 expression. *Fgfr3-GFP^{+}* femur at P21 with GREM1 staining (upper three panels). *Fgfr3-creER*; *R26R*^{tdTomato} femur at P23 (pulsed at P21) with GREM1 staining (lower three panels). Scale bar: 20µm. *n*=4 mice per each group.
Data are presented as mean ± s.d. Source data are provided as a Source Data file.

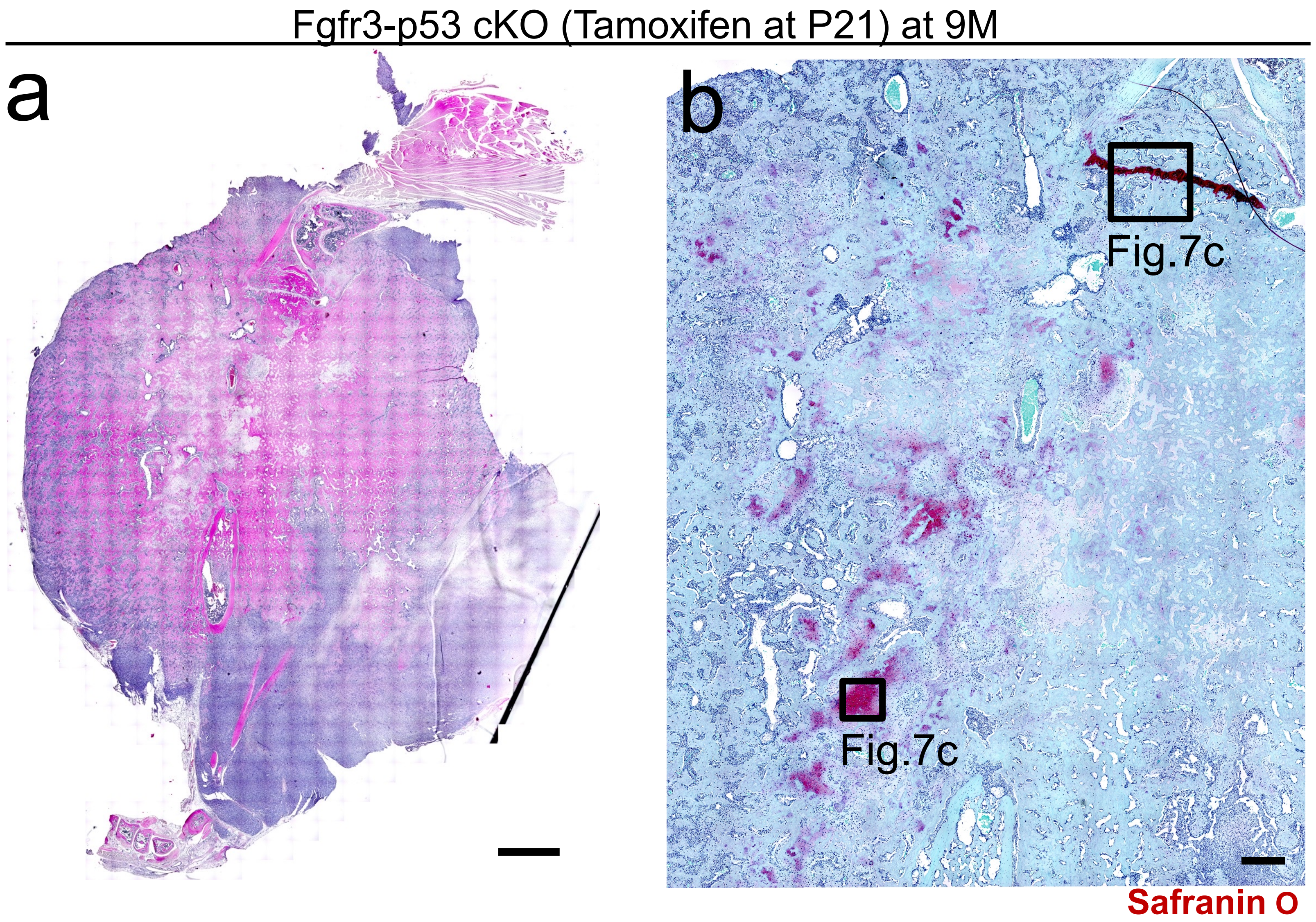

Supplementary Figure 7. Fgfr3⁺ endosteal stromal cells develop aggressive osteosarcoma-like lesions upon p53 loss

(a,b) Osteosarcoma-bearing *Fgfr3-creER; Trp53$^{fl/fl}$; R26R^{tdTomato}* hind limb (pulsed at P21) at 9M. *n*=11 mice. (a): H&E staining. Scale bar: 2mm. (b): Chondroid matrix within osteosarcoma-like neoplastic lesion. Safranin O staining. Scale bar: 500 µm (left).

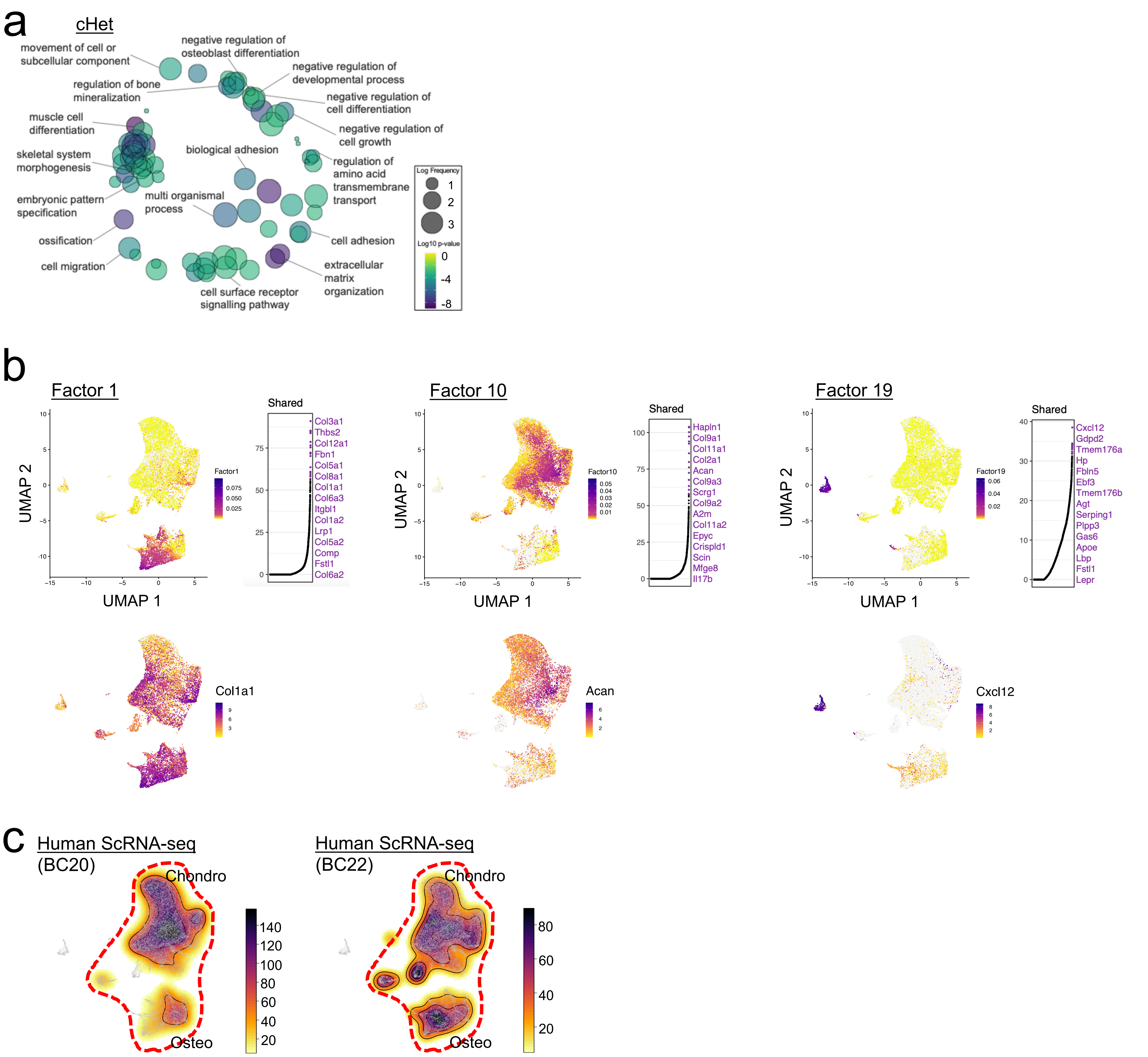

Supplementary Figure 8. Single-cell characterization of Fgfr3⁺ cell-derived mouse osteosarcoma-like lesions

(a) Gene Ontology term enrichment analysis for cHet by REVIGO. REVIGO plot of gene ontology terms enriched among genes upregulated in putative tumor cells. The p-values were calculated using GOrilla's one-sided test based on the hypergeometric distribution, with a flexible p-value cutoff applied for multiple testing correction. The results were subsequently transferred to REVIGO for visualization.
(b) Plots showing loading values of metagene (top) and marker gene (bottom) which specifically loads on Osteo, Chondro, and Reticular clusters. Top: plots showing metagene (factor)-loading values for each cell type (UMAP plots) and shared gene loadings (on a particular metagene; dot plots) for factor 1, 10, and 19, which specifically loads on Osteo, Chondro, and Reticular clusters. In gene-loading plots, gene names are sorted in decreasing order of magnitude of their factor-loading contribution and correspond to colored points in scatter plots. The gene names in purple represent the top-loading genes. Bottom: UMAP representations of gene loading values for *Col1a1* (Osteo), *Acan* (Chondro), and *Cxcl12* (Reticular).
(c) LIGER data integration of single-cell RNA-seq datasets of mouse and human osteosarcoma cells. Density plots of human tumor cells (left: BC20, right: BC22) in the integrated space.

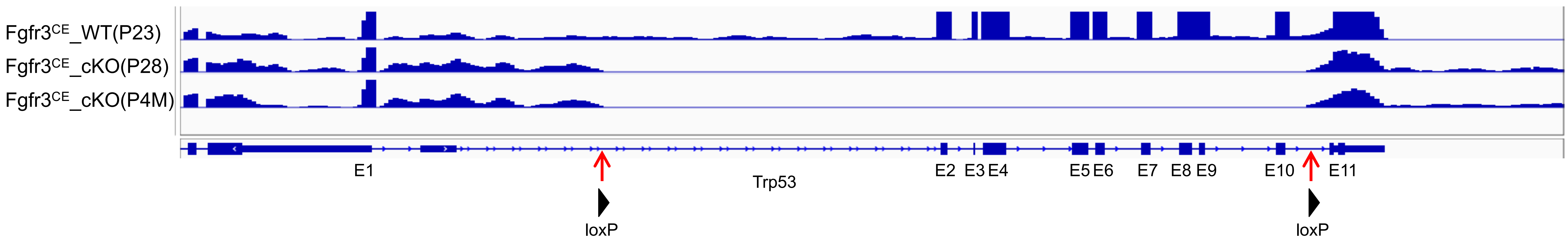

Supplementary Figure 9. Unregulated self-renewal and aberrant osteogenic fates of tumorigenic Fgfr3+ stem cells.

Structure of the genomic *Trp53* locus and the *loxP* sites in the *Trp53* floxed allele. Bulk RNA-seq based mRNA levels of *Trp53* exons in Fgfr3-WT (P23), Fgfr3-p53 cKO (P28) and Fgfr3-p53 cKO (P4M) tdTomato+ cells.
